# Simukunin from the Salivary Glands of the Black Fly *Simulium vittatum* Inhibits Enzymes That Regulate Clotting and Inflammatory Responses

**DOI:** 10.1371/journal.pone.0029964

**Published:** 2012-02-23

**Authors:** Hitoshi Tsujimoto, Michail Kotsyfakis, Ivo M. B. Francischetti, Jai Hoon Eum, Michael R. Strand, Donald E. Champagne

**Affiliations:** 1 Department of Entomology, The University of Georgia, Athens, Georgia, United States of America; 2 Center for Tropical and Emerging Global Diseases, The University of Georgia, Athens, Georgia, United States of America; 3 Laboratory of Genomics and Proteomics of Disease Vectors, Institute of Parasitology, Biology Center of the Academy of Sciences of Czech Republic, Ceske Budejovice, Czech Republic; 4 Laboratory of Malaria and Vector Research, National Institute of Allergy and Infectious Diseases, National Institutes of Health, Rockville, Maryland, United States of America; Johns Hopkins School of Public Health, United States of America

## Abstract

**Background:**

Black flies (Diptera: Simuliidae) feed on blood, and are important vectors of *Onchocerca volvulus*, the etiolytic agent of River Blindness. Blood feeding depends on pharmacological properties of saliva, including anticoagulation, but the molecules responsible for this activity have not been well characterized.

**Methodology/Principal Findings:**

Two Kunitz family proteins, SV-66 and SV-170, were identified in the sialome of the black fly *Simulium vittatum*. As Kunitz proteins are inhibitors of serine proteases, we hypothesized that SV-66 and/or −170 were involved in the anticoagulant activity of black fly saliva. Our results indicated that recombinant (r) SV-66 but not rSV-170 inhibited plasma coagulation. Mutational analysis suggested that SV-66 is a canonical BPTI-like inhibitor. Functional assays indicated that rSV66 reduced the activity of ten serine proteases, including several involved in mammalian coagulation. rSV-66 most strongly inhibited the activity of Factor Xa, elastase, and cathepsin G, exhibited lesser inhibitory activity against Factor IXa, Factor XIa, and plasmin, and exhibited no activity against Factor XIIa and thrombin. Surface plasmon resonance studies indicated that rSV-66 bound with highest affinity to elastase (*K_D_* = 0.4 nM) and to the active site of FXa (*K_D_* = 3.07 nM). We propose the name “Simukunin” for this novel protein.

**Conclusions:**

We conclude that Simukunin preferentially inhibits Factor Xa. The inhibition of elastase and cathepsin G further suggests this protein may modulate inflammation, which could potentially affect pathogen transmission.

## Introduction

Both eukaryotes and prokaryotes produce Kunitz family protease inhibitors, which indicates an ancient origin for Kunitz family encoding genes [1], [2]. The most conserved function of Kunitz family proteins is the reversible competitive inhibition of serine proteases [1]. A single Kunitz domain is small (∼60 aa) and forms a compact globular fold typically containing three disulfide bonds. Based on the structure of bovine pancreatic trypsin inhibitor (BPTI), a typical Kunitz domain contains cysteine residues at positions 5, 14, 30, 38, 51 and 55 in the mature peptide, which form three disulfide bonds C5–C55, C14–C38 and C30–C51 [1]. The Kunitz domain may exist singly, or as multiple domains within a single polypeptide [3]. Many Kunitz domains act as protease inhibitors through their scissile bond at positions 15 (P_1_) and 16 (P_1_′). The P_1_ residue is often a basic amino acid (K or R) while the P_1_′ position is an A or G, which together interact with the active site of one or more proteases [1]. Alternative modes of action have also been characterized. For example, snake venoms contain Kunitz family proteins named dendrotoxins that exhibit weak anti-protease activity but strongly block neuronal K^+^ channels [4].

Blood coagulation in mammals is a physiological response that is activated by a complex enzymatic cascade, consisting primarily of serine proteases, and which terminates with formation of a fibrin clot. Negative regulators of coagulation are primarily protease inhibitors, which include one Kunitz family protein named tissue factor pathway inhibitor (TFPI) [5] that inhibits formation of FXa by binding to the FVIIa-Tissue factor-FXa complex [3], [5]. Blood-feeding arthropods also produce anti-hemostatic factors in their saliva, which facilitate blood feeding by interfering with host hemostatic responses [6]. A variety of anti-coagulation factors have been identified from arthropods including several Kunitz family proteins in the saliva of ticks [3], [7]. For example, ixolaris from the deer tick, *Ixodes scapularis*, contains two kunitz domains. The N-terminal Kunitz displays a glutamic acid residue in the P_1_ position, while the C-terminal Kunitz atypically has only 4 cysteines [8]. Ixolaris binds to the heparin-binding exosite of coagulation Factor X (FX) and FXa through the C-terminal domain, and this complex forms a tight-binding inhibitor of the FVIIa/Tissue Factor complex. The saliva of *I. scapularis* also contains penthalaris, which has five Kunitz domains and inhibits the tissue factor pathway in a manner similar to ixolaris [9]. Other Kunitz family proteins from tick saliva exhibit functions that range from anti-thrombin and anti-FXa activity to anti-kallikrein and anti-platelet aggregation [3], [10].

Black flies (Diptera: Simuliidae) like *Simulium vittatum* are small, stout-bodied insects. Females of *S. vittatum* and most other species must feed on blood from a vertebrate host to produce multiple clutches of eggs. Black flies are not only a nuisance for humans and livestock but vector several pathogens including *Onchocerca volvulus* that causes onchocerciasis, (river blindness) in humans, and vesicular stomatitis virus that causes disease in livestock. The bites of *S. vittatum* induce a pronounced and persistent erythema [11] due to the presence of a salivary protein named *S. vittatum* erythema protein (SVEP) [12]. *S. vittatum* saliva also contains at least three anti-coagulation factors, which exhibit activity against thrombin, FXa, or FV [13]–[16]. The identity of these anti-hemostatic factors, however, remains unknown.

A recent publication on the combined transcriptome and proteome (collectively called the “sialome”) of *S. vittatum* salivary glands detected many transcripts and corresponding tryptic peptide fragments including two Kunitz family proteins, named SV-66 and SV-170, that could function as anti-coagulation factors [17]. In this study, we expressed SV-66 and SV-170 and assessed their anti-coagulant activity. Our results indicated that SV-66 is an anti-coagulant with anti-FXa activity that also inhibits several other serine proteases.

## Results

### 2.1. SV-66 and SV-170 encode conserved Kunitz proteins

**Figure 1:**
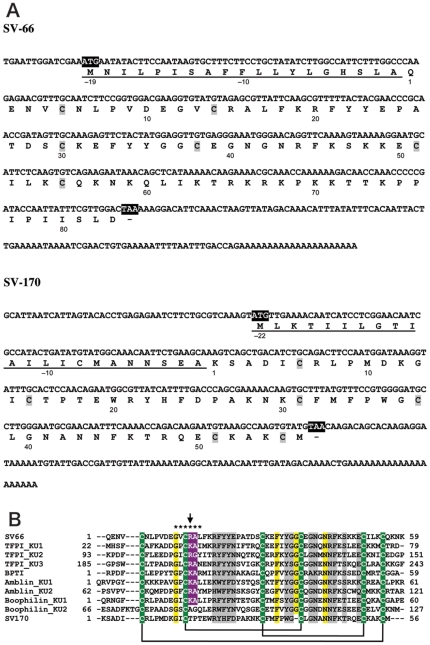
SV-66 and SV-170 belong to the Kunitz family of protease inhibitors. (A) Nucleotide and translated polypeptide sequences of SV-66 and SV-170. Start and stop codons are in white with black shading. Numbers below the amino acid residues are designated based on the putative mature protein. Signal sequences predicted by SignalP are underlined. Top: SV-66 encodes a 102 amino-acid polypeptide (Simukunin), which includes a 19 amino-acid N-terminal signal sequence. Mature Simukunin is predicted to consist of 83 amino-acid residues, with a theoretical mass of 9627.22 Da and pI of 9.93. SV-66 also contains a putative O-glycosylation site at position 81 (Ser). Bottom: SV-170 encodes a 78 amino-acid polypeptide, which includes an N-terminal 22 amino-acid signal sequence. Mature SV-170 is predicted to consist of 56 amino-acid residues, and theoretical mass and pI are 6526.66 Da and 8.87, respectively. (B) Alignment of representative Kunitz domain sequences with SV-66 and SV-170. Each Kunitz domain was separated from the original sequences for alignment (numbers denote amino-acid positions in the original mature peptides). All reference sequences were retrieved from GenBank. Accession numbers are: TFPI (human: 3 Kunitz domains), P10646; BPTI (*Bos taurus*: 1 Kunitz domain), AAI49369; Amblin (*Amblyomma hebraeum*: 2 Kunitz domains), AAR97367; Boophilin (*Rhipicephalus microplus*: 2 Kunitz domains), CAC82583. Strictly conserved cysteine residues are white with green shading, and predicted conserved disulfide bonds are shown in solid lines. The reactive site loop (RSL) P_4_-P_2_′ residues, conserved in canonical binding inhibitors, are indicated by asterisks. The P_1_ residue is indicated with an arrow. Highly conserved P_1_-P_1_′ (Arg/Lys-Ala/Gly) residues are shown in white with purple shading. Other identical residues across the domain are shaded with yellow and conserved or semi-conserved residues are shaded with grey.

SV-66 and SV-170 consist of 309 and 237 nucleotides respectively that encode predicted proteins of 102 and 78 amino acids (Figure 1A). SignalP identified signal sequences for SV-66 and SV-170 of 19 and 22 amino acids respectively. We assigned residue numbers based on the predicted mature proteins and indicated signal sequence residues as negative numbers (Figure 1A). Alignment with selected other Kunitz-domain containing proteins indicated that SV-66 and SV-170 possess six conserved cysteine residues and other conserved residues characteristic of Kunitz family members (Figure 1B). SV-66 exhibited a basic arginine residue at position 15, which was the predicted P_1_ residue. This finding suggested that SV-66 may be an active protease inhibitor. In contrast, SV-170 had a threonine at the predicted P_1_ position, which suggested a lack of a canonical inhibitory activity against trypsin-like serine proteases, but which was similar to the C-terminal Kunitz domain of boophilin [18].

### 2.2. SV-66 is expressed in female salivary glands

**Figure 2:**
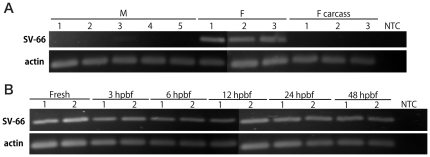
SV-66 is constitutively expressed in the salivary glands of adult female *S. vittatum*. (A) Sex and tissue-specific expression of Simukunin. Transcript was detected in the adult female body (head and thorax without abdomen), but not in adult female carcasses (bodies without salivary glands or heads). M: male; F: mature female; NTC: no-template control. 5 individuals were pooled for each sample. Actin PCR products are shown as a positive control indicating equivalent concentrations of template among samples. Each panel is a composite of two rows (upper and lower) of wells, run in the same gel at the same time. (B) Time-course of expression of Simukunin. Transcript was detected before, and at selected time points up to 48 h post blood meal. Fresh: freshly eclosed non-blood-fed female adult; hpbf: hours post blood feeding; NTC: no-template control. Two samples, each comprised of 5 pooled individuals, were analyzed for each time point.

We performed RT-PCR assays to qualitatively assess SV-66 expression in adult *S. vittatum*. We detected amplicons of expected size in cDNA samples prepared from the female body (head and thorax) but did not detect any amplicons from female carcasses (female body minus salivary glands and head) or adult males (Fig. 2A). Time course studies indicated that SV-66 was expressed by adult females prior to blood feeding as well as at all time points we sampled after blood feeding (Fig. 2B). Taken together, these results suggested that SV-66 was constitutively expressed in the salivary glands of adult females but was not expressed in males.

### 2.3. rSV-66 inhibits plasma clotting, while rSV-170 does not

We cloned SV-66 and SV-170 cDNAs into the pET-30 Ek/LIC vector (Novagen) and expressed each in *Escherichia coli* as recombinant (r) proteins with C-terminal double His tags. Two-step purification using Ni^2+^ resin and RP-HPLC yielded fractions highly enriched for rSV-66 or rSV-170 (Figure S1).

**Figure 3:**
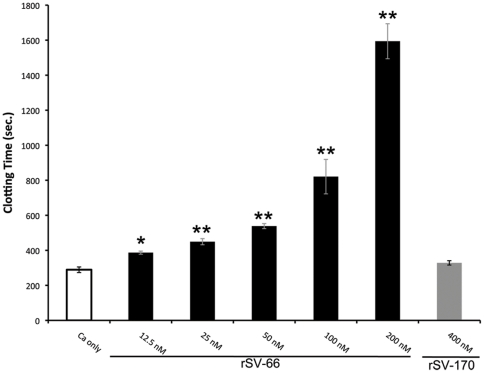
rSV-66 delays clotting of human plasma. Indicated concentrations of rSimukunin (rSV-66) and rSV-170 were tested by the recalcification time assay. Citrated human plasma (50 µl) was mixed with recombinant proteins (in 50 µl 0.15 M NaCl, 10 mM HEPES pH7.4) and pre-warmed at 37°C for 15 min before clotting was initiated by the addition of 50 µl prewarmed CaCl_2_ (25 mM). Recalcification (clotting) time was determined by monitoring absorbance at 650 nm at 10-sec intervals in a SpectraMax 340 microtiter plate reader, with onset time (the time to a linear increase in the OD, which reflects the maximal rate of formation of insoluble fibrin) set at an OD of 0.04. Clotting times (mean ± SD) for rSimukunin and rSV-170 are shown in black bars and grey bars, respectively. The white bar is the Ca^2+^-only control. One-way analysis of variance indicted a significant difference between treatments (F_6, 21_ = 119.3; P<0.001) for rSimukunin but not rSV-170. Subsequent multiple comparisons between various treatments and the positive control were performed using the Holm-Sidak method. Statistically significant increases in clotting time at *p*<0.05 and *p*<0.01 are indicated by * and ** respectively. Results shown are representative of three independent experiments.

We examined the effect of rSV-66 and rSV-170 on the time needed for fibrin deposition from Ca^2+^-stimulated normal human plasma (clotting time) by measuring the increase in OD at 650 nm. rSV-66 prolonged coagulation time in a dose-dependent manner, beginning at concentrations as low as 12.5 nM, whereas rSV-170 exhibited no anti-coagulation activity up to a concentration of 400 nM (Figure 3). Since rSV-170 did not inhibit coagulation, we focused the remainder of the study on rSV-66. Given its anticoagulant activity, we named rSV-66 “Simukunin”, after a contraction of *Simulium* kunitz inhibitor. We also used the name Simukunin and rSimukunin for recombinant Simukunin during the remainder of this study.

### 2.4. Residues in the reactive site loop are important for rSimukunin anti-coagulation activity

**Figure 4:**
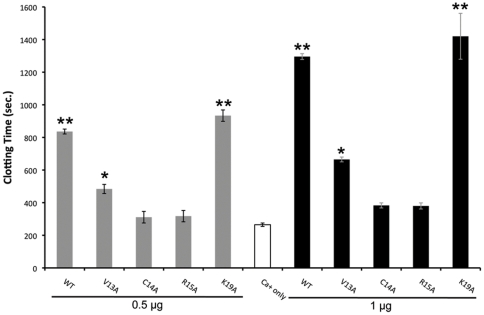
Point mutations in the reactive site loop of rSimulkunin disable anti-coagulation activity. Each recombinant protein was tested by adding 0.5 µg (grey bars) or 1 µg (black bars) to a fixed volume of plasma. Plasma was then pre-warmed at 37°C for 15 min before addition of 25 mM (8.3 mM final concentration) CaCl_2_ (pre-warmed) to initiate clotting. White bar shows the Ca^2+^-only plasma control. The graph shows mean ± SD from three independently conducted experiments. *p*-values for significant differences by one-tailed t-test are shown, where the alternative hypothesis is that sample recalcification time is greater than the Ca^2+^-only control. Statistically significant increases in clotting time at *p*<0.05 and *p*<0.01 are indicated by * and ** respectively.

Since canonical Kunitz inhibitors interact with target proteases through their reactive site loop (RSL), we asked whether alanine replacement of residues in or adjacent to the RSL affected Simukunin activity by producing three alanine replacement mutants, SV66^V13A^, SV66^C14A^, and SV66^R15A^. We also produced a fourth mutant, SV66^K19A^, as a control protein with an alteration outside of the RSL domain that we hypothesized should not affect anticoagulation activity. Following purification by Ni^2+^ affinity resin and reversed-phase HPLC, we observed that each recombinant protein ran as a doublet on SDS-PAGE gels (not shown). Tryptic digestion and mass spectrometry analysis, however, indicated that both bands consisted solely of Simukunin that were identical to full length native Simukunin. This analysis also indicated that the low molecular-weight band corresponded to loss of one of the epitope tags, possibly due to cleavage by an *E. coli* protease during the lysis step. We thus concluded the presence of this lower band should not affect the RSL or activity. However, its presence necessitated the use of µg rather than molar concentrations in coagulation assays since precise calculation of molar concentrations was impossible. We therefore conducted anticoagulation assays by adding 0.5 or 1.0 µg of WT or mutant rSimukunin to plasma and compared the rapidity of clotting to plasma without rSimukunin by pairwise t-test. The presence of WT rSimukunin significantly increased clotting time as did addition of SV66^K19A^. In contrast, the addition of SV66^C14A^ and SV66^R15A^ to plasma had no significant effect on clotting activity. SV66^V13A^ delayed coagulation, but this effect was reduced compared to the delay produced by WT rSimukunin (Figure 4).

### 2.5. rSimukunin inhibits activity of multiple proteases including coagulation factors

**Figure 5:**
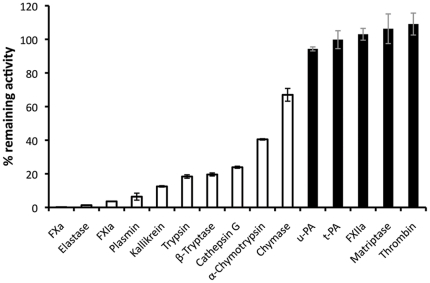
rSimukunin inhibits several serine proteases. Enzyme activity in the presence of 500 nM of rSimukunin, as a percentage of the total activity in the absence of inhibitor, is shown. Results with statistically significant (t-test, *p*<0.01) inhibition compared to the control are shown in white bars. The graph shows mean ± SEM. u-PA: urokinase-type plasminogen activator, t-PA: tissue plasminogen activator. Each inhibition assay was conducted in triplicate.

Preliminary assays indicated that rSimukunin also inhibited the enzymatic activity of enterokinase, which suggested rSimukunin could inhibit other serine proteases besides those with roles in host coagulation. We therefore characterized the inhibitory activity of rSimukunin against 15 different serine proteases that included the coagulation factors FXa, FXIa, FXIIa, and thrombin. Our results indicated that rSimukunin significantly reduced the activity of ten of these proteases (Figure 5). In the coagulation cascade, rSimukunin significantly inhibited FXa and FXIa, but not thrombin or FXIIa. Other enzymes strongly inhibited by rSimukunin were elastase, plasmin, kallikrein, trypsin, β-tryptase, and cathepsin G (Figure 5).

We determined the IC_50_ of rSimukunin to the most strongly inhibited proteases in order to assess the relative affinity of this inhibitor for each target (Table 1). To obtain linear reaction rates within the time frame of the experiment, we assayed each target enzyme at different molar concentrations. We therefore also calculated the molar ratio of rSimukunin to the enzyme at the IC_50_ (Table 1). Collectively, our results indicated that rSimukunin most strongly inhibited elastase with an IC_50_ of 4.9 nM and a ratio of inhibitor to enzyme of 27.22. Factor Xa was also strongly inhibited, with an IC_50_ of 5.2 nM and a molar ratio of 52.00, as was cathepsin G with a molar ratio of 32.45. The other enzymes tested, including FXIa, required molar excesses of hundreds- to thousands-fold for 50% inhibition of proteolytic activity.

**Table 1:** IC_50_ values for rSimukunin against various serine proteases.

Enzyme	Concentration (nM)	IC_50_ (mean ± SEM (nM)	Ratio
Elastase	0.18	4.9±0.6	27.22
Cathepsin G	6.7	217.4±5.7	32.45
Factor Xa	0.1	5.2±0.3	52.00
Plasmin	0.2	32.2±5.2	161.00
Factor XIa	0.06	56.7±13.1	945.00
Kallikrein	0.08	91.8±6.3	1147.50
Trypsin	0.1	379.3±30.2	3793.00
β-Tryptase	0.01	66.8±14.6	6680.00

Titrated concentrations of rSimukunin were tested with constant concentrations of enzymes (in Concentration column) to determine the concentrations of rSimukunin that gave a 50% inhibition of the enzyme activity. Ratios of IC_50_ to enzyme concentration are also shown as different concentrations of enzymes were necessary to obtain linear reaction rates. Titration curves are shown in Figure S2.

As elastase, FXa, and cathepsin G were the most strongly inhibited, based on the ratio of inhibitor to enzyme (Table 1), we determined the *K_m_* for these three enzymes, and then used the approach of Cheng and Prusoff [19] to calculate the *K_i_* from the IC_50_, with a final substrate concentration of 250 uM. The *K_m_* for the elastase substrate was 38.5±3.3 µM, and the estimated *K_i_* was∼0.65 nM. For FXa the substrate *K_m_* was 161.6±17.4 µM, and the estimated *K_i_* was∼2.1 nM. For Cathepsin G the *K_m_* was 188±20 µM, and the *K_i_* was 100 nM. We used a general chymotrypsin substrate to assess Cathepsin G activity; as this substrate is not optimized for Cathepsin G it is likely that our measurement of the *K_i_* underestimates the effect of rSimukunin. Further studies of the effect of rSimukunin on Cathepsin G are planned.

### 2.6. rSimukunin displays high affinity binding to FXa and elastase

**Figure 6:**
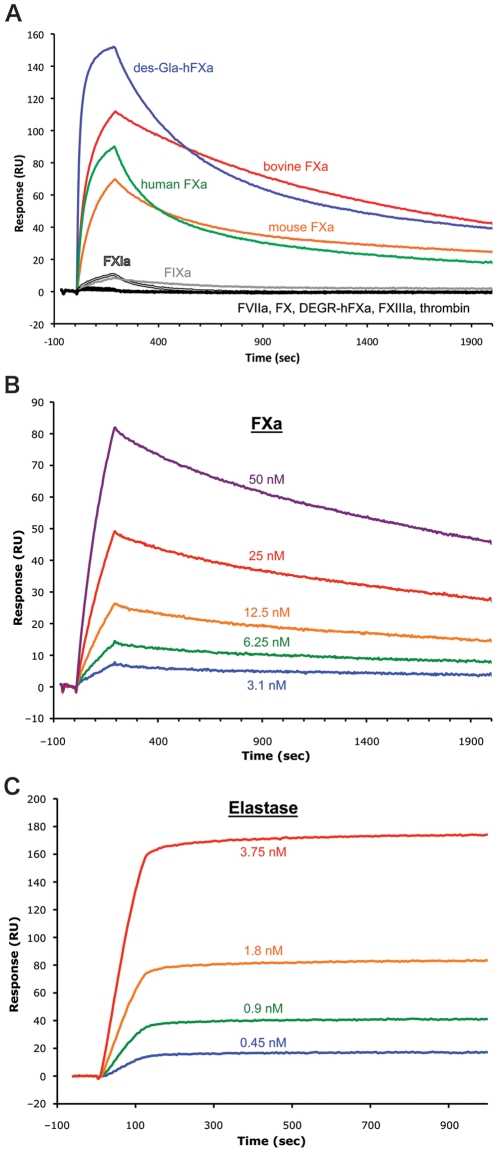
rSimukunin displays high-affinity binding to FXa and elastase. (A) Surface Plasmon Resonance (SPR) Sensorgrams show mouse, bovine, and human FXa, the FXa derivatives des-GLA-hFXa and DEGR-hFXa, and other coagulation factors (all tested at 200 nM) binding to immobilized rSimukunin. (B) Sensorgrams for various concentrations of human FXa (in nM: purple, 50; red, 25; orange, 12.5; green, 6.25, blue, 3.1) binding to immobilized rSumukunin. (C) Sensorgrams for various concentrations of elastase (in nM: red, 3.75; orange, 1.8; green, 0.9; blue, 0.45) binding to immobilized rSimukunin. Data were fitted using a 1∶1 binding model (Langmuir). RU: resonance units.

The preceding results provided measures of affinity for elastase, FXa, and cathepsin G by rSimukinun, but were insufficient for calculation of binding and dissociation kinetics. We therefore conducted Surface Plasmon Resonance (SPR) studies using rSimukunin immobilized on a sensor chip and used selected target enzymes as the analyte. These studies showed that rSimukunin bound FXa from several mammals (human, mouse and bovine) with high affinity (Figure 6A). rSimukunin also bound DES-Gla-FXa, an FXa derivative lacking the Gla domain necessary for docking onto a negatively charged membrane surface. In contrast, rSimukunin did not bind DEGR-FXa, a derivative blocked at the active site, or FX, which is the zymogen precursor of FXa (Figure 6A). rSimukunin also exhibited very weak binding responses to FIXa and FXIa, and no binding responses to FVIIa, FXIIIa or thrombin (Figure 6A). Kinetic analysis determined that FXa bound rSimukunin with a *K_D_* of 3.07 nM (Figure 6B and Table 2), but assays with elastase revealed an even stronger affinity for rSimukunin with a *K_D_* = ∼0.4 nM (Figure 6C and Table 2). These *K_D_* values are consistent with the *K_i_* values we calculated based on the *K_m_* and IC_50_. The low *K_D_* values for both FXa and elastase result from a fast association rate (*ka1* = 4.3×10^7^ M^−1^ s^−1^ for elastase) and a very slow off rate (*kd1* = 0.017 s^−1^) indicative of rSimukunin functioning as a tight-binding inhibitor.

**Table 2:** Kinetics of FXa and Elastase interactions with rSimukunin.

Enzyme	*ka1* (M^−1^ s^−1^)	*kd1* (s^−1^)	*K_D_* (nM)
FXa	9.87×10^4^	3.032×10^−4^	3.071
Elastase	4.302×10^7^	0.01727	0.40

Responses were obtained by injecting FXa or elastase over immobilized rSimukunin for 180 seconds at a flow rate of 30 µl/minute. Data were derived from *Ka1* and *Kd1* and fitted using the Langmuir (1∶1 binding) equation. Kinetic values obtained from the sensorgrams presented in Figure 6B and 6C for FXa and elastase respectively.

## Discussion

Salivary gland extracts from *S. vittatum* have long been known to have potent anticoagulant activity, but the identity of the molecules involved had heretofore remained uncharacterized. In the current study, we show that of the two Kunitz family proteins expressed in the salivary glands of female *S. vittatum* [17], rSV-66, which we name Simukunin, dose-dependently prolongs coagulation times of human plasma while rSV-170 lacks anticoagulant activity. Simukunin is constitutively expressed specifically in adult female salivary glands, which is consistent with a role in blood feeding.

Our SPR results strongly suggest that the anticoagulant activity of Simukunin is primarily due to high affinity binding to FXa. In contrast, rSimukinin does not bind to the zymogenic form of FX, which suggests this inhibitor only interacts with the activated enzyme. Our results further show that rSimukunin exhibits binding to des-Gla-FXa but does not interact with DEGR-FXa, which is blocked specifically at the active site. These data together with outcomes of our mutagenesis studies strongly suggest that rSimukunin interacts directly with the active site of FXa. That FXa is a primary target of rSimukunin is also supported by our results showing that rSimukunin does not interact with FVIIa, FXIIa, FXIIIa, or thrombin, and only weakly interacts with FIXa and FXIa.

The loss of anticoagulation activity by SV66^R15A^ is fully consistent with the hypothesis that rSimukunin interacts directly with the active site of FXa, and that Arg15 functions as the P_1_ residue. The reduced activity of SV66^V13A^ could likewise reflect a less stable interaction with the catalytic active site of FXa, while the lack of activity of SV66^C14A^ likely reflects an alteration in structure due to elimination of the Cys14–Cys38 disulfide bond. Taken together, we think it likely that Simukunin acts similarly to BPTI, whose basic P_1_ residue, Lys15, is critical for the inhibition of trypsin and chymotrypsin [20] through stable docking into the S_1_ specificity subsite of these enzymes and formation of polar interactions with a negatively charged Asp189 side chain [1].

The anti-clotting activity of Simukunin may account for the anti-FXa activity of black fly SGE described previously [13]. However, the estimated size of a partially characterized FXa inhibitor was 18000 Da [13], which is much larger than the predicted size of Simukunin (9627.22 Da). Although Simukunin has a putative O-glycosylation site in the C-terminal region, the 1D SDS-PAGE gel band from which Simukunin tryptic fragments were recovered migrated between the 14.1 and 6 kDa markers in Andersen *et al*. [17], which indicates that glycosylation does not significantly add to the native size of Simukunin. The relationship between Simukunin and the previously described anti-FXa activity thus remains unclear but it is possible that other proteins contribute to the anti-FXa activity in black fly saliva.

Although rSimukunin inhibited kallikrien in our screening assay, the activity was modest, and further, it did not inhibit bradykinin production from kaolin-activated human plasma (JMC Ribeiro, pers. Commun. May 21, 2010), indicating that the FXII/prekallidrein/kallikrein pathway is not a target of this inhibitor.

Coagulation and inflammation are linked through a variety of cross-talk mechanisms [21]. For example, proteinase-activated receptor 4, which is highly expressed on platelets, is activated following cleavage by FXa or thrombin and subsequently mediates inflammatory responses [21], [22]. As Simukunin inhibits FXa, which indirectly reduces subsequent thrombin activation, it is likely that Simukunin may also influence inflammatory responses at the bite site, though we have not yet examined this experimentally.

Our comparative data clearly show that rSimukunin binds to and/or inhibits several other enzymes besides FXa including major components of neutrophil azurophil granules such as elastase, and mast cell proteases including cathepsin G, tryptase, and chymase. Indeed, the strongest binding affinity we detected was between rSimukunin and elastase. This finding together with inhibition of cathepsin G is notable because both enzymes function in killing phagocytized microbes by neutrophils. Extracellular release of cathepsin G mediates platelet aggregation, which is known to provide a surface for assembly of the prothrombinase complex [21]. Thus, inhibition of cathepsin G could indirectly antagonize coagulation. Cathepsin G, tryptase, and elastase also regulate the function of several chemokines, cytokines, cell surface receptors and adhesion molecules [23]–[26], which leads to the possibility that Simukunin may affect inflammation or other responses in proximity to sites of black fly feeding. Further experimental work will be necessary to examine the effect of Simukunin on macrophage, mast cell, and neutrophil function.

The inhibition of elastase by rSimukunin is somewhat surprising in light of prior studies showing that Kunitz domain inhibitors of elastase like Bikunin (formerly called acid stable trypsin inhibitor or ASTI, or urinary trypsin inhibitor) have an aliphatic amino acid, such as methionine, leucine, or valine in the P_1_ position [27]–[30]. We thus speculate that residues outside the RSL either orient the Simukunin/elastase interaction to permit tight binding despite the unfavorable P_1_ residue, or that binding and inhibition involves interactions with a domain distinct from the RSL. Future structure-function experiments, however, will be required to understand how Simukunin interacts with this enzymatic target.

Inhibition of multiple enzymes by rSimukunin, is also not unusual for Kunitz family inhibitors from blood-feeding arthropods and other organisms. For example, the archetypical Kunitz protein BPTI inhibits trypsin with a *K_i_* of 0.06 pM, but it also inhibits chymotrypsin (*K_i_* = 9 nM), plasmin, and kallikrein [31]. Boophilin, a tick salivary protein with two Kunitz domains, likewise exhibits significant inhibitory activity toward thrombin, trypsin, plasmin, and plasma kallikrein [18], while the serpin IRS-2 has inhibitory activity against cathepsin G, chymase, and a-chymotrypsin [32]. On the other hand, Kunitz family thrombin inhibitors from soft ticks appear to specifically inhibit thrombin at pM concentrations (eg. ornithodorin, *K_i_* = 1 pM [33]; savignin, *K_i_* = 4.89 pM) [34]. Lastly, we note that the binding affinities of Simukunin for FXa (*K*_D_ = 3.071 nM) and elastase (*K*_D_ = 0.4 nM) are broadly consistent with the binding affinities determined for other Kunitz inhibitors from blood feeding species. As examples, the tick salivary inhibitor ixolaris binds to FX and FXa with *K_D_*s of 0.67 and 0.25 nM respectively [35], while boophilin binds thrombin with a *K_i_* of 1.8 nM [18], as does amblin, with a *K_i_* of 20 nM [36].

Future functional studies will be needed to fully characterize the role of Simulkunin in blood feeding. Nonetheless, our results collectively suggest Simukunin facilitates blood-feeding by disrupting coagulation and possibly by interfering with host inflammatory responses. Several examples from mosquitoes and sandflies also implicate saliva in potentiating pathogen transmission by modulating immune responses in the host skin (reviewed in [37]–[39]). However, future studies will be required to determine whether Simukunin affects pathogen transmission by black flies. Lastly, we note that recent studies of the sialome from another black fly, *Simulium nigrimanum*, identified four Kunitz protein-encoding transcripts, which are divided into two subfamilies based on the presence or absence of a C-terminal extension of basic residues [40]. Therefore, *S. nigrimanum* salivary glands may also produce multiple Kunitz anti-coagulation factors with potential roles in regulating coagulation and inflammation.

## Materials and Methods

### 4.1. *S. vittatum* culture

*S. vittatum* was reared at the Department of Entomology, the University of Georgia, according to previously described conditions [41], [42]. Because *S. vittatum* is facultatively autogenous, no vertebrate animals were used in the study for rearing or maintenance of insects.

### 4.2. Salivary gland dissection, cDNA synthesis, and expression analyses

Adult female flies (collected 2–3 days post eclosion) were chilled on ice, and their salivary glands were collected by dissection under a stereomicroscope in sterile HEPES saline (HS) (10 mM HEPES pH 7.0, 150 mM NaCl). Fifty salivary gland pairs were collected in 20 µL HS, mixed with 20 µL RNA*later* (Ambion, Foster City, CA), and stored at –70°C until use. Total RNA was isolated using Trizol reagent (Invitrogen, Carlsbad, CA). Genomic DNA was removed using RNase-free DNase (Turbo DNase, Ambion, Foster City, CA). First strand cDNA was synthesized using 100 ng of total RNA, SuperScript III (Invitrogen, Carlsbad, CA) and an oligo d(T) primer (5′-(T)_25_-VN-3′) at 50°C for 60 min, followed by enzyme inactivation at 70°C for 15 min. Resultant cDNA was then stored at −20°C until use.

Total RNA was isolated and reverse transcribed as described above from female flies without salivary glands and head (termed “carcass”), intact female flies, and intact males flies. Since *S. vittatum* is autogenous for the first gonotrophic cycle, we also reverse transcribed RNA from female bodies without abdomens (in order to avoid collecting vertebrate cells in the blood meal) collected after the first oviposition, and at 3, 6, 12, 24, and 48 hrs post blood-feeding (pbf). Blood-fed flies were kept at 27°C with constant access to sugar water (10% sucrose). RT-PCRs were then run using an Eppendorf thermocycler in 25 µl reaction volumes containing 1 µl of cDNA and 2.5 µM of SV-66 UA and SV-66 DA primers (Table S1). Cycling conditions were as follows: initial 2 min denaturation at 94°C, followed by 30 cycles at 30 sec at 94°C, 30 sec at 57°C 30 sec at 72°C, and a final 5 min extension at 72°C. Actin from *S. vittatum* (GenBank accession number AY083375.1) amplified using SVactinUA and SVactinDA primers (Table S1) was used as an endogenous control.

### 4.3. Cloning and sequence analysis of Kunitz family proteins

Full-length cDNAs for SV-66 and SV-170 (GenBank accession numbers EU930300 and EU930227, respectively) [17] were amplified from salivary gland cDNA using Platinum HIFI Taq DNA polymerase and gene specific primers (SV66 UA, SV66DA, SV170UA, SV170DA) in 25 µl reactions. PCR conditions were: initial 2 min denaturation at 94°C, followed by 35 cycles (30 sec at 94°C, 30 sec at 57°C 30 sec at 68°C), and a final 5 min extension at 68°C. The resulting amplicons were then TA cloned into pCR4-TOPO (Invitrogen), and transformed into *E. coli* TOP10 competent cells (Invitrogen). After overnight culture, the plasmids SV66/TOPO and SV170/TOPO were purified using the Wizard *Plus* miniprep DNA purification system (Promega, Madison, WI) and quantified using a NanoDrop spectrophotometer (Thermo Scientific, Wilmington, DE). Inserts were sequenced (Macrogen, Rockville, MD) to confirm fidelity with the reference sequences. SignalP [43] was used to predict signal peptide sequences, and NetOGlyc [44] was used to predict potential glycosylation sites. Alignments were performed using Clustal W2 [45] against other Kunitz domains from the following Kunitz inhibitors: bovine pancreatic trypsin inhibitor (BPTI) (Genbank accession number AAI49369) from *Bos taurus* (a single Kunitz domain inhibitor); human tissue factor pathway inhibitor (TFPI) (P10646) (consisting of 3 Kunitz domains); a thrombin inhibitor, amblin, (AAR97367) from the tick *Amblyomma hebraeum* (2 Kunitz domains); and a prothrombinase inhibitor, boophilin, (CAC82583) from the tick *Rhipicephalus* (*Boophilus*) *microplus* (2 Kunitz domains).

### 4.4. Protein expression and purification

For bacterial expression of SV-66 and SV-170, full-length ORFs without signal peptides were PCR amplified using SV66/TOPO or SV170/TOPO as template, the primers SV66UB/SV66DB or SV170UB/SV170DB (Table S1), and Elongase polymerase enzyme mix (Invitrogen). The resulting products were then directly cloned in frame with the C-terminal His-tag of the vector pET-30 (Novagen) using T4 polymerase. We generated the mutants SV66^V13A^, SV66^C14A^, SV66^R15A^, and SV66^K19A^ using the Quick Change site-directed mutagenesis kit (Strategene) together with the primers V13A-UA/V13A-DA, C14A-UA/C14A-DA, R15A-UA/R15A-DA, and K19A-UA/K19A-DA respectively. Each of these constructs were confirmed by DNA sequencing, and then expressed by transforming into *E. coli* BL21 (DE3) cells cultured in SOC medium (0.5% Yeast Extract, 2% Tryptone, 10 mM NaCl, 2.5 mM KCl, 10 mM MgCl_2_, 10 mM MgSO_4_, 20 mM Glucose), supplemented with 10 ug/ml of kanamycin to an O.D. of 1.0 at 37°C. We then added 0.1 mM isopropyl-β-d-thiogalactopyranoside (IPTG) to the cultures and grew an additional 17–24 h at 20°C. Bacterial cells were harvested by centrifugation at 4500×*g* for 10 min and used immediately or stored at −80°C.

Bacterial pellets from 0.8 L cultures were resuspended in 40 ml of lysis buffer (50 mM Tris-HCl pH 8.0, 300 mM NaCl, 10 mM imidazole). After addition of lysozme (1 mg/ml) in 50 mM Tris-HCl (pH 8.0), cells were incubated on ice for 1 h followed by two freeze-thaw cycles and sonication with six, 10 sec bursts at 300 W using a Branson 450 Sonifier (VWR). The lysate was then centrifuged at 13,000×*g* for 10 min, followed by 3 h incubation of the supernatant with Ni-NTA Superflow beads (Qiagen) pre-equilibrated with lysis buffer. After washing, attached proteins were eluted with three column volumes of elution buffer (50 mM Tris-HCl pH 8.0, 300 mM NaCl, 300 mM imidazole), followed by desalting and concentration using an Amicon Ultra-4 3000 MWCO spin column (Millipore, USA). Proteins were quantified using the Micro BCA Protein Assay Kit (Pierce) and visualized after SDS-polyacrylamide gel electrophoresis (PAGE) (4–20% precast gels (Lonza)) by staining with Coomassie Blue.

Recombinant proteins were further purified by reversed-phase high-performance liquid chromatography (RP-HPLC) using a Jupiter C4 column (5 µm particle size, 300 Å pore size, 250 mm length×2.00 mm ID) (Phenomenex, Torrance, CA) with a linear gradient between 95% H_2_O/5% acetonitrile (ACN)/0.05% trifluoroacetic acid (TFA)) and 95% ACN/5% H_2_O/0.03% TFA) monitored at 220 nm. Fractions were collected every minute, lyophilized to remove ACN and TFA, resuspended in 20 mM Tris pH 8.0, and quantified by the BCA method described above. Purity of the proteins was assessed by SDS-PAGE, performed as described above, and immunoblotting. Separated proteins were transferred onto a polyvinylidene fluoride (PVDF) membrane (Bio Rad, Hercules, CA), blocked in 1× PBS containing 0.05% Tween-20 and 2% non-fat skim milk for 1 hour at room temperature, and incubated with an anti-His primary antibody (His-probe (H-15); Santa Cruz Biotechnology) (1∶5000). The membrane was washed three times with 1× PBS containing 0.05% Tween-20, incubated with goat anti-rabbit secondary antibody conjugated to horseradish peroxidase (Jackson Immuno Research) (1∶10000), and visualized by chemiluminescence using the ECL Plus Western Blotting system (GE Healthcare, Piscataway, NJ).

### 4.5. Recalcification assay

Recalcification assays were performed as described by Valenzuela et al [46]. Briefly, 10 µL of various concentrations of recombinant proteins, 40 µL of 0.15 M NaCl, 10 mM HEPES pH7.4 and 50 µL of citrated human plasma (TriniCHECK Level 1, Trinity Biotech, Co Wicklow, Ireland) were mixed in a flat-bottomed 96-well plate and prewarmed at 37°C for 15 min. To initiate plasma clotting, 50 µL of 25 mM CaCl_2_ prewarmed to 37°C was added in the 96-well plate. Immediately after the addition of CaCl_2_ absorbance was taken at 650 nm at 10-sec intervals by microtiter plate reader at 37°C (SpectraMax 340, Molecular Devices, Sunnyvale, CA). Clotting onset time (“Clotting Time”) was set at the time when the absorbance reached an optical density (OD) of 0.04, where the increase in OD is linear. One-way ANOVA, followed by pairwise comparisons of treatment values to the CaCl_2_-only control using the Holm-Sidak method, was used for the statistical analysis; when *p*<0.05, the difference was considered as statistically significant.

### 4.6. Serine protease inhibition assays

rSimukunin (500 nM) was first pre-incubated with each enzyme for 10 min before the addition of the corresponding substrate. The amount of enzyme used in the assays was the lowest possible to give a linear substrate hydrolysis rate in the assays (r^2^>0.95). After incubation for 5 min at 30°C, substrate hydrolysis rate was followed in a Tecan Infinite M200 96-well plate fluorescence reader (Tecan group Ltd, Switzerland) using 365 nm excitation and 450 nm emission wavelength with a cutoff at 435 nm for 20 min at 30°C. Wells without enzyme were used to monitor spontaneous substrate hydrolysis and protease contamination in the inhibitor preparation. A t-test was used for the statistical analysis of the observed inhibition in the presence of 500 nM rSimukunin and when *p*<0.05, it was considered as statistically significant.

IC_50_ estimates were determined as previously described [47], using decreasing concentrations of rSimukunin pre-incubated for 5 min with a given target enzyme, followed by addition of substrate. All experiments were performed in triplicate (for each enzyme and each concentration of the inhibitor). The mean percentage of enzymatic activity in the presence of various rSimukunin concentrations was then compared with enzymatic activity in the absence of rSimukunin. The sigmoidal fit of the data then yielded the estimate for the IC_50_ of rSimukunin for the various enzymes. The resulting titration curves are provided in Figure S2.

All enzymes used were of human origin and of the highest available purity. The source and concentration of the enzymes used in the serine protease screen assays follows: Thrombin (0.02 nM), α-chymotrypsin (0.025 nM), plasmin (0.2 nM) and chymase (1.8 nM) were purchased from Sigma (St. Louis, MO), skin β-tryptase (0.01 nM) was purchased from Promega (Madison, WI), activated coagulation factor X (FXa) (0.1 nM) was purchased from EMD Biosciences (La Jolla, CA), FXIIa (0.1 nM) was purchased from Haematologic Technologies Inc. (Essex Junction, VT), kallikrein (0.08 nM) was purchased from Fitzgerald Industries International (Concord, MA), elastase (0.18 nM) was purchased from Elastin Products (Owensville, MO), FXIa (0,06 nM), urokinase-type plasminogen activator (u-PA) (0.5 nM) and tissue plasminogen activator (t-PA) (0.06 nM) from Molecular Innovations (Southfield, MI), matriptase (0.2 nM) from R&D Systems (Minneapolis, MN), cathepsin G (6.7 nM) from Enzo Life Sciences (Plymouth Meeting, PA) and sequencing grade trypsin (0.1 nM) was purchased from Roche (Chicago, IL). The amount of enzyme used in each of the IC50 estimation assays is shown also in the Table 1.

Assay buffers were: 1) for elastase and chymase, 50 mM HEPES buffer pH 7.4, 100 mM NaCl, 0.01% Triton X-100; 2) for trypsin, α-chymotrypsin, factor XIa, factor XIIa and thrombin, 50 mM Tris buffer pH 8.0, 150 mM NaCl, 20 mM CaCl_2_, 0.01% Triton X-100; 3) for β-tryptase, 50 mM Tris pH 8.0, 50 mM NaCl, 0.05% Triton X-100; 4) for kallikrein, matriptase and plasmin, 20 mM Tris buffer pH 8.5, 150 mM NaCl, 0.02% triton X-100; 5) for factor Xa, 20 mM Tris buffer pH 8.0, 200 mM NaCl, 5 mM CaCl_2_, 0.1%BSA; 6) for u-PA and t-PA, 20 mM Tris buffer pH 8.5, 0.05% Triton X-100; 7) for cathepsin G, 50 mM Tris buffer pH 7.4, 150 mM NaCl, 0.01% Triton X-100. Peptidyl substrates used were: Suc-A-A-P-V-AMC for elastase, α-chymotrypsin and chymase (EMD Biosciences, La Jolla, CA); Boc-D-P-R-AMC for thrombin and plasmin; Boc-Q-A-R-AMC for trypsin, factor XIa and u-PA (Sigma, St. Louis, MO); Boc-F-S-R-AMC for β-tryptase; Suc-L-L-V-Y-AMC for chymase (Bachem, King of Prussia, PA); and methylsulfonyl-D-cyclohexylalanyl-G-R-AMC acetate for factor Xa, factor XIIa, t-PA, matriptase and kallikrein (American Diagnostica Inc., Stamford, CT). All substrates were used at 250 µM final concentration in all the assays.

### 4.7. Surface plasmon resonance (SPR) assays

SPR experiments were conducted using a T100 instrument (Biacore Inc., Uppsala, Sweden) following the manufacturer's instructions. Sensor CM5, amine coupling reagents, and buffers were also purchased from Biacore Inc. (Piscataway, NJ, USA). HBS P (10 mM Hepes, pH 7.4, 150 mM NaCl, and 0.005% (v/v) P20 surfactant) was used as the running buffer for all SPR experiments, which were carried out as previously described [28]. For analytes, FVIIa (recombinant) was purchased from American Diagnostica (Stamford, CT); FX, FXa (human, bovine, mouse), DEGR-FXa (human), des-Gla-FXa (human), FIXa, FXIa, FXIIIa, and thrombin were purchased from Haematologic Technologies, Inc. (Essex Junction, VT); elastase (purified from human sputum) was purchased from Molecular Innovations, Inc. (Novi, MI). All enzymes were of the highest available purity. For immobilization and kinetic analysis, rSimukunin (10 µg/ml) in acetate buffer pH 5.0 was immobilized over a CM5 sensor via amine coupling, resulting in a final immobilization of 475.3 RU. Kinetic experiments were carried out with a contact time of 180 seconds at a flow rate of 30 µl/minute at 25°C. rSimukunin-FXa complex dissociation was monitored for 1800 seconds, and the sensor surface was regenerated by a pulse of 30 seconds of 20 mM HCl at 30 µl/minute. In other experiments, other coagulation factors or enzymes were used as analytes. Blank flow cells were used to subtract the buffer effect on sensorgrams. After subtraction of the contribution of bulk refractive index and nonspecific interactions with the CM5 chip surface, the individual association (*k_a_*) and dissociation (*k_d_*) rate constants were obtained by global fitting of data to a 1∶1 interaction model (Langmuir) using BIAevaluation™ (Biacore, Inc.) software [48]:
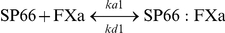
Values were then used to calculate the dissociation constant (*K_D_*). Conditions were chosen so that the contribution of mass transport to the observed values of *K_D_* was negligible. Also, models in the T100 evaluation software fit for mass transfer coefficient to mathematically extrapolate the true *ka* and *kd*.

## Supporting Information

Figure S1 **Visualization of rSV-66 and rSV-170 following separation by SDS-PAGE (left panel) and immunoblotting (right panel).** The SDS-PAGE gel was stained with Coomassie Brilliant Blue, while the immunoblot was probed with an anti-His primary antibody and visualized by chemiluminescence. Lanes were loaded with bacterial lysate (L), Ni^2+^ resin purified protein, (Ni^2+^), or protein further purified by RP-HPLC (HPLC). Western blotting of the bacterial lysates was done separately from the Ni^2+^ and RP-HPLC purified proteins. The figure is therefore a composite, with the lysate lanes aligned with the others based on protein standards, but blotting protocols were identical for all samples.
(TIF)

Figure S2 **Determination of rSimukunin IC_50_ values for selected serine proteinases.** Enzymes, at the concentrations given in Table 1, were incubated with the indicated concentration of rSimukunin for 5 min at 30°C, followed by addition of substrate (250 µM final concentration). The amount of enzyme used in the assays was the lowest possible to give a linear substrate hydrolysis rate in the assays (r^2^>0.95). Substrate hydrolysis was followed in a *Tecan* Infinite M200 96-well plate fluorescence reader (*Tecan* group Ltd, Switzerland) using 365 nm excitation and 450 nm emission wavelength with a cutoff at 435 nm for 20 min at 30°C. Wells without enzyme were used to monitor spontaneous substrate hydrolysis and protease contamination in the inhibitor preparation. All experiments were performed in triplicate (for each enzyme and each concentration of the inhibitor). The mean percentage of enzymatic activity in the presence of various rSimukunin concentrations was then compared with enzymatic activity in the absence of rSimukunin. The sigmoidal fit of the data then yielded the estimate for the IC_50_ of rSimukunin for the various enzymes reported in Table 1.
(TIF)

Table S1 **PCR primers used in this study.** For primers used for cloning in pET-30, direction-specific LIC sites are underlined. For primers used for single His-tag constructs, bold letters indicate the stop codon (TAA) and the read-through Ala (GCA in reverse-complement orientation).
(DOC)
