# Supplementary figures and images for: Simukunin from the Salivary Glands of the Black Fly Simulium vittatum Inhibits Enzymes That Regulate Clotting and Inflammatory Responses
Source: PLoS One. 2012 Feb 23;7(2):e29964. doi: 10.1371/journal.pone.0029964 (PMC3285612; doi:10.1371/journal.pone.0029964)

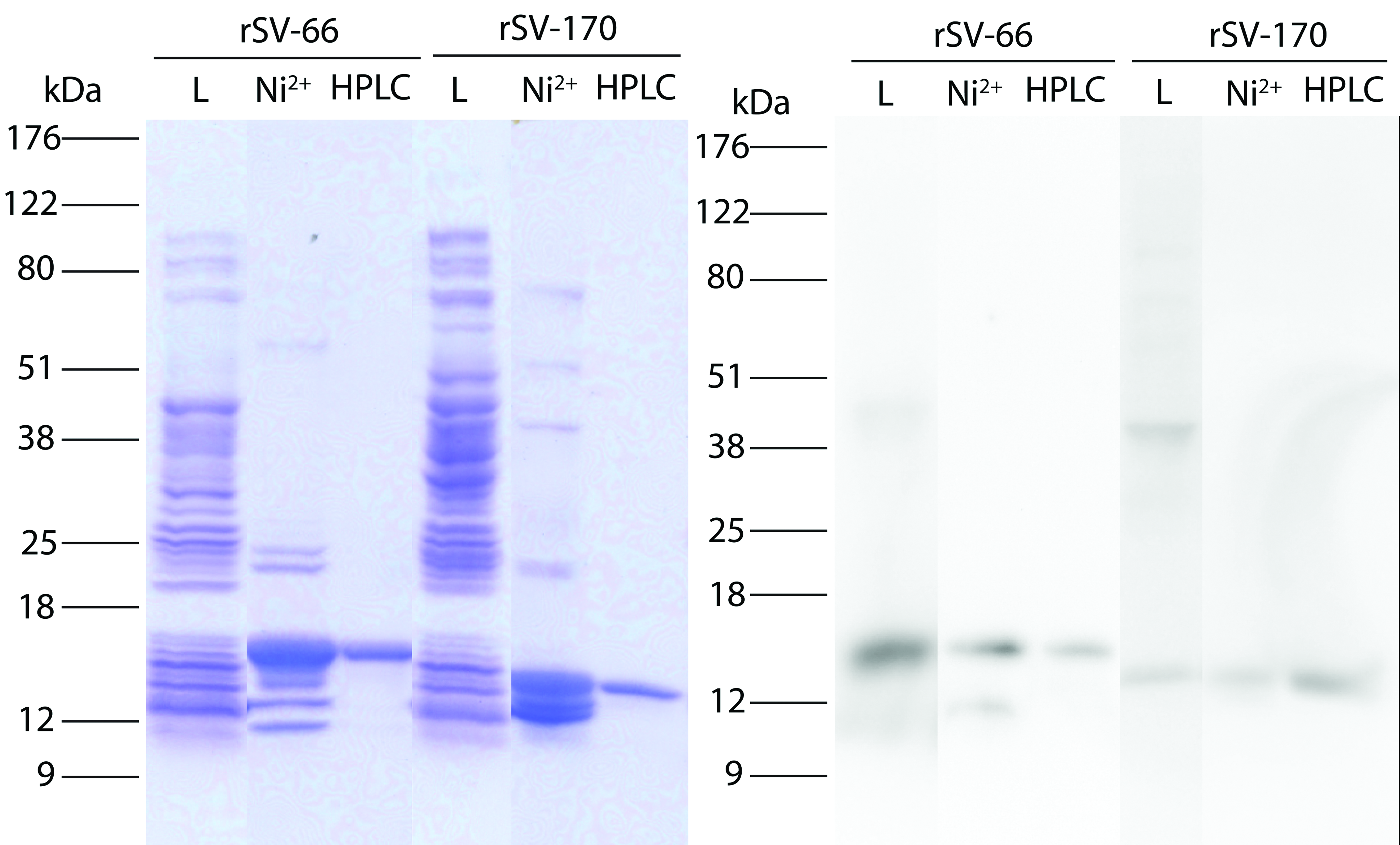

Supplement: Figure S1 — Visualization of rSV-66 and rSV-170 following separation by SDS-PAGE (left panel) and immunoblotting (right panel). The SDS-PAGE gel was stained with Coomassie Brilliant Blue, while the immunoblot was probed with an anti-His primary antibody and visualized by chemiluminescence. Lanes were loaded with bacterial lysate (L), Ni2+ resin purified protein, (Ni2+), or protein further purified by RP-HPLC (HPLC). Western blotting of the bacterial lysates was done separately from the Ni2+ and RP-HPLC purified proteins. The figure is therefore a composite, with the lysate lanes aligned with the others based on protein standards, but blotting protocols were identical for all samples. (TIF) [file pone.0029964.s001.tif]

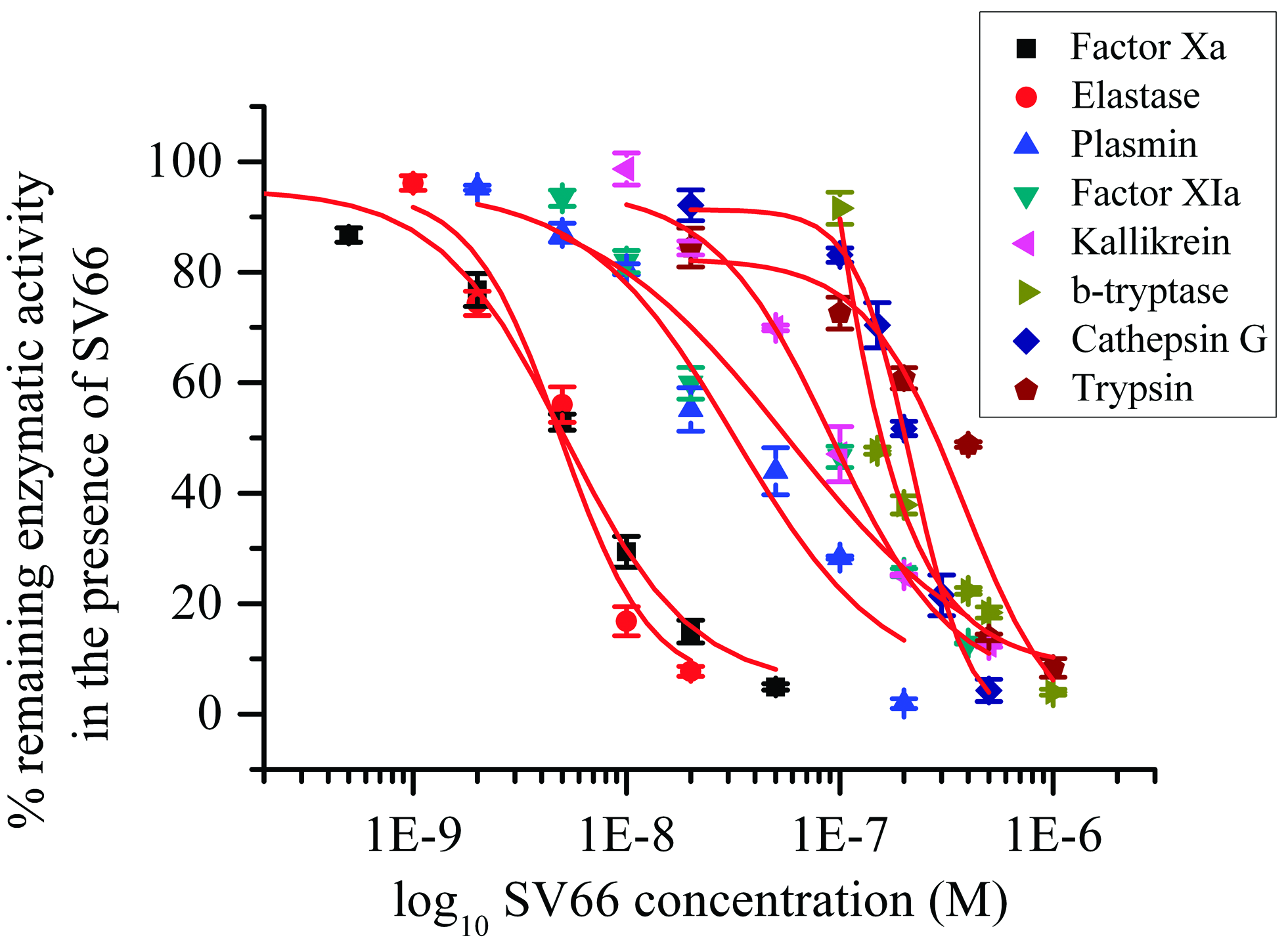

Supplement: Figure S2 — Determination of rSimukunin IC50 values for selected serine proteinases. Enzymes, at the concentrations given in Table 1, were incubated with the indicated concentration of rSimukunin for 5 min at 30°C, followed by addition of substrate (250 µM final concentration). The amount of enzyme used in the assays was the lowest possible to give a linear substrate hydrolysis rate in the assays (r2>0.95). Substrate hydrolysis was followed in a Tecan Infinite M200 96-well plate fluorescence reader (Tecan group Ltd, Switzerland) using 365 nm excitation and 450 nm emission wavelength with a cutoff at 435 nm for 20 min at 30°C. Wells without enzyme were used to monitor spontaneous substrate hydrolysis and protease contamination in the inhibitor preparation. All experiments were performed in triplicate (for each enzyme and each concentration of the inhibitor). The mean percentage of enzymatic activity in the presence of various rSimukunin concentrations was then compared with enzymatic activity in the absence of rSimukunin. The sigmoidal fit of the data then yielded the estimate for the IC50 of rSimukunin for the various enzymes reported in Table 1. (TIF) [file pone.0029964.s002.tif]
