# Supplementary material for: Simukunin from the Salivary Glands of the Black Fly Simulium vittatum Inhibits Enzymes That Regulate Clotting and Inflammatory Responses
Source: PLoS One. 2012 Feb 23;7(2):e29964. doi: 10.1371/journal.pone.0029964 (PMC3285612; doi:10.1371/journal.pone.0029964)
Supplement: Table S1 — PCR primers used in this study. For primers used for cloning in pET-30, direction-specific LIC sites are underlined. For primers used for single His-tag constructs, bold letters indicate the stop codon (TAA) and the read-through Ala (GCA in reverse-complement orientation). (DOC) [file pone.0029964.s003.doc]

| Primer | Sequence (5’ → 3’) |
| --- | --- |
| SVactinUA | TGTGTTACGTTGCCTTGGACTTTG |
| SVactinDA | TGATGGAGTTGTAGACGGTTTCGTG |
| SV66UA | TGAATTGGATCGAAATGAATATACTTCCA |
| SV66DA | TTAGTTTGAATGTCCTTTTTAGTCCAACGA |
| SV170UA | CACCTGAGAGAATCTTCTGCGTCAAA |
| SV170DA | CGGTCAATACATTTTTATCCTCTTGTGCT |
| SV66UB | GACGACGACAAGATGCAAGAGAACGTTTGCAATCTTC |
| SV66DB | GAGGAGAAGCCCGG**TTA**GTCCAACGAAATAATTGGTATC |
| SV66DC | GAGGAGAAGCCCGG**TGC**GTCCAACGAAATAATTGGTATCG |
| SV170UB | GACGACGACAAGATGAAGTCAGCTGACATCTGCAGA |
| SV170DB | GAGGAGAAGCCCGG**TTA**CATACACTTGGCTTTACATTCT |
| SV170DC | GAGGAGAAGCCCGG**TGC**CATACACTTGGCTTTACATTCTTG |
| V13A-UA | CCGGTGGACGAAGGTGCATGTAGAGCGTTATTC |
| V13A-DA | GAATAACGCTCTACATGCACCTTCGTCCACCGG |
| C14A-UA | AATCTTCCGGTGGACGAAGGTGTAGCTAGAGCGTTATTCA |
| C14A-DA | TGAATAACGCTCTAGCTACACCTTCGTCCACCGGAAGATT |
| R15A-UA | CTTCCGGTGGACGAAGGTGTATGTGCAGCGTTATTCAAGC |
| R15A-DA | GCTTGAATAACGCTGCACATACACCTTCGTCCACCGGAAG |
| K19A-UA | GGTGTATGTAGAGCGTTATTCGCGCGTTTTTACTACGAACCC |
| K19A-DA | GGGTTCGTAGTAAAAACGCGCGAATAACGCTCTACATACACC |
